# Dual Plating of the Distal Femur: Indications and Surgical Techniques

**DOI:** 10.7759/cureus.6483

**Published:** 2019-12-27

**Authors:** Arnab Sain, Vijay Sharma, Kamran Farooque, Muthukumaran V, Kirubakaran Pattabiraman

**Affiliations:** 1 Orthopaedics, All India Institute of Medical Sciences, New Delhi, IND

**Keywords:** dual-plating, distal femur, medial plate fixation

## Abstract

Dual-plating of the distal femur is required in some cases to achieve stable fixation. The indications of a medial plate in addition to the lateral plate are medial supracondylar bone loss, low trans-condylar bicondylar fractures, medial Hoffa fracture, peri-prosthetic distal femur fractures, non-union after failed fixation with single lateral plate, poor bone quality and comminuted distal femur fractures (AO type C3). We recommend orthogonal plate configuration with locked plates by a single incision or dual incision approach as per surgeon choice.

## Introduction and background

Supracondylar femur fractures are commonly associated with severe comminution and significant soft tissue injury. Distal femoral fractures are mostly caused by high-energy injuries, such as falling injury and traffic accidents, and fractures are often severely comminuted. Despite the recent advances in techniques and implants, the treatment of intra-articular multi-fragmentary distal femoral fractures remains a challenge. Long-term disability can occur in patients with extensive articular cartilage damage and marked comminution. Distal femur fractures in the elderly are complicated by poor bone quality (severe osteoporosis), a distal segment that is too short for adequate fixation, blood loss, malunion and non-union, and increased mortality [1-4].

Locked plating is one of the best and modern options for treating supracondylar femur fractures with relatively low failure rates. Single lateral plating of distal femur fractures was often found to have a relatively higher failure rate [2].

A medial plate in addition to lateral plating reduces the chances of failure of fixation [2]. This article will focus on the indications and techniques of using an additional medial plate fixation in fractures of the distal femur.

## Review

Pre-operative planning

Decision making before surgical stabilization is important. The treating surgeon should examine the injured limb to check for the soft tissue status and any distal neurovascular deficit. Also, other injuries should be ruled out. Good quality radiographs in two planes are necessary. Computed tomography of the distal femur with coronal and sagittal reformations helps in delineating fracture patterns. Proper pre-operative planning will guide towards the need for dual plating of distal femur fractures [5].

Indications for dual plating

Supracondylar femur fractures were previously treated with condylar buttress plates [6]. Subsequently, fixed-angle implants, angle-blade plates, intramedullary retrograde nails, and dynamic supracondylar screws were found to have a better biomechanical design for preventing the varus collapse compared to condylar buttress plates [7,8]. Locking plates are known for having increased biomechanical resistance with the advantage of greater numbers of fixation screws in the distal femur metaphysis [9,10]. Locking plates provide increased stability and resistance to failure compared to retrograde nails in elderly patients with poor bone stock [11-13]. The additional application of a medial plate in the following circumstances may help in achieving a stable anatomically aligned reduction.

Medial Supracondylar Bone Loss

**Figure 1:**
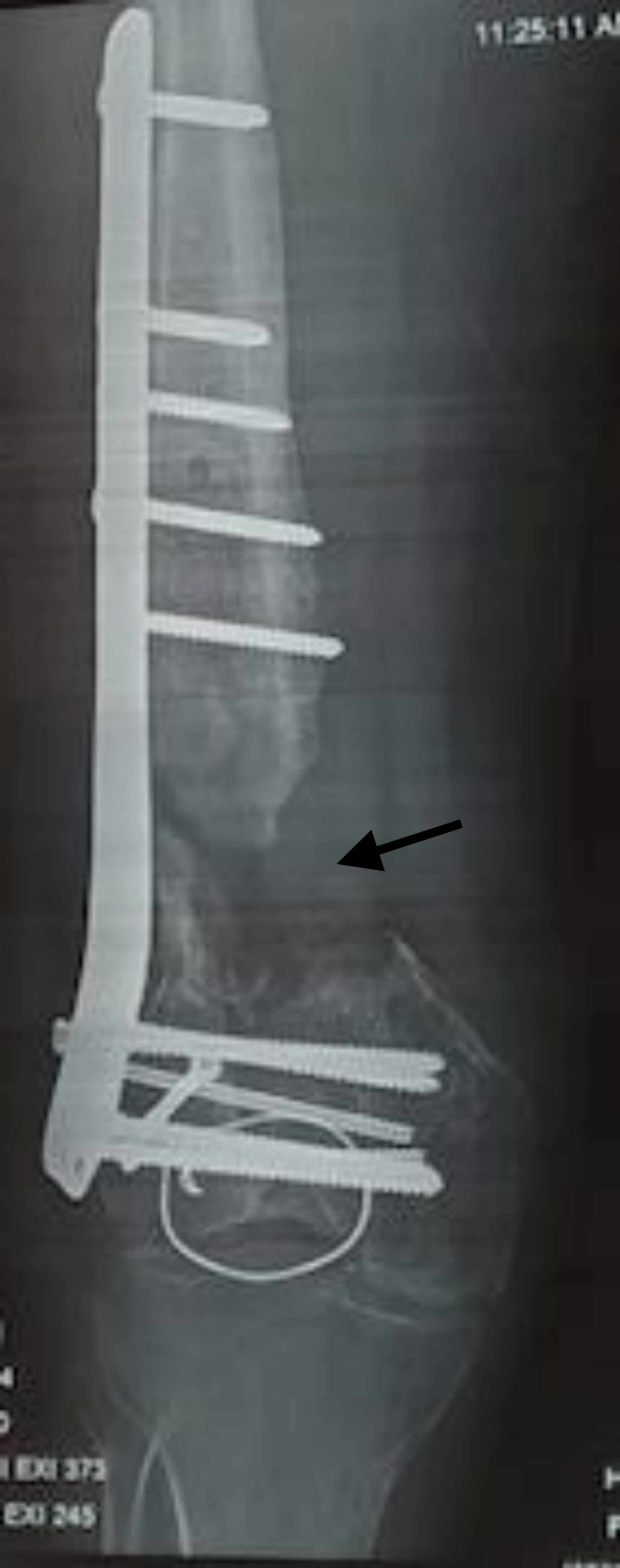
Failure of fixation with a single lateral plate in a 55-year-old patient with an open fracture and medial supracondylar bone loss. Note the medial bone loss of more than 2 cm.

**Figure 2:**
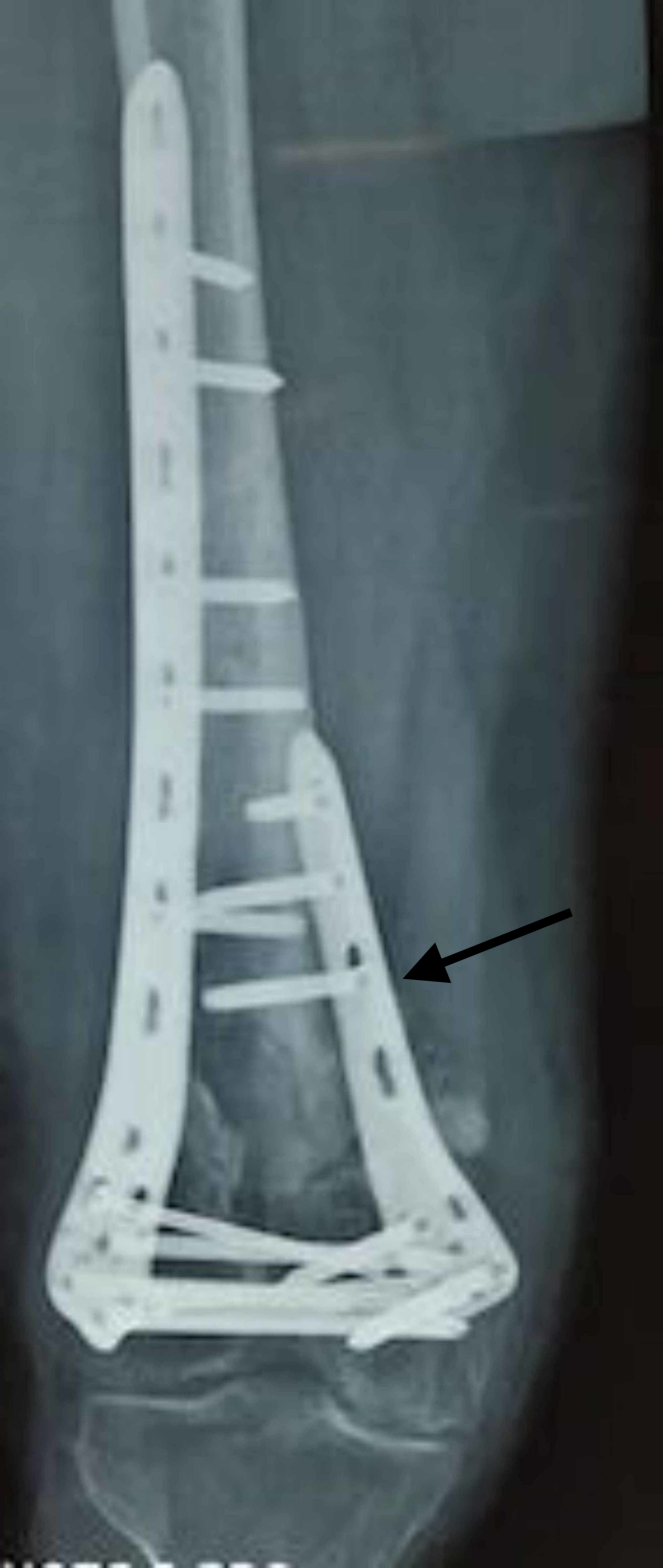
Revision with a medial plate and bone grafting.

In distal femur fractures with extensive metaphyseal comminution, osteopenic bone and in high-energy or open fractures, there is functional loss of medial cortical buttress. In these situations, there is less chance of healing of the medial column. The addition of a medial plate helps in giving additional stability and reduces the chances of implant failure [1,14]. Rajasekaran et al. found that additional medial plating and bone grafting was needed for non-union distal femur with medial bone loss more than 2 cm (Figures 1, 2) [15].

Low Trans-condylar Bicondylar Fractures

High energy injuries sometimes lead to a low horizontal trans-condylar fracture pattern which is associated with an intercondylar split. In this fracture pattern, fixation with a single lateral plate may not achieve stable fixation as sufficient screw purchase will not be possible because of the intercondylar notch. A medial buttress plate in addition to the lateral plate is helpful in this situation [5,16].

Medial Hoffa Fracture

Anatomical restoration of the articular surface is necessary for comminuted distal femur fractures. In fractures with a large medial Hoffa fragment, the application of a medial neutralization plate is necessary to achieve stable fixation. This fixation provides stability in addition to interfragmentary lag screws [5].

Peri-prosthetic Distal Femur Fractures

In peri-prosthetic fractures of the distal femur, dual plating offers stable fixation, especially in osteoporotic bone. This allows for early mobilization and rehabilitation. Stable fixation of peri-prosthetic fractures is necessary to avoid failures of fixation. Cicek et al. advocated dual locked plate fixation of peri-prosthetic distal femur fractures with osteoporotic bones [17].

Non-union after Failed Fixation with Single Lateral Plate

**Figure 3:**
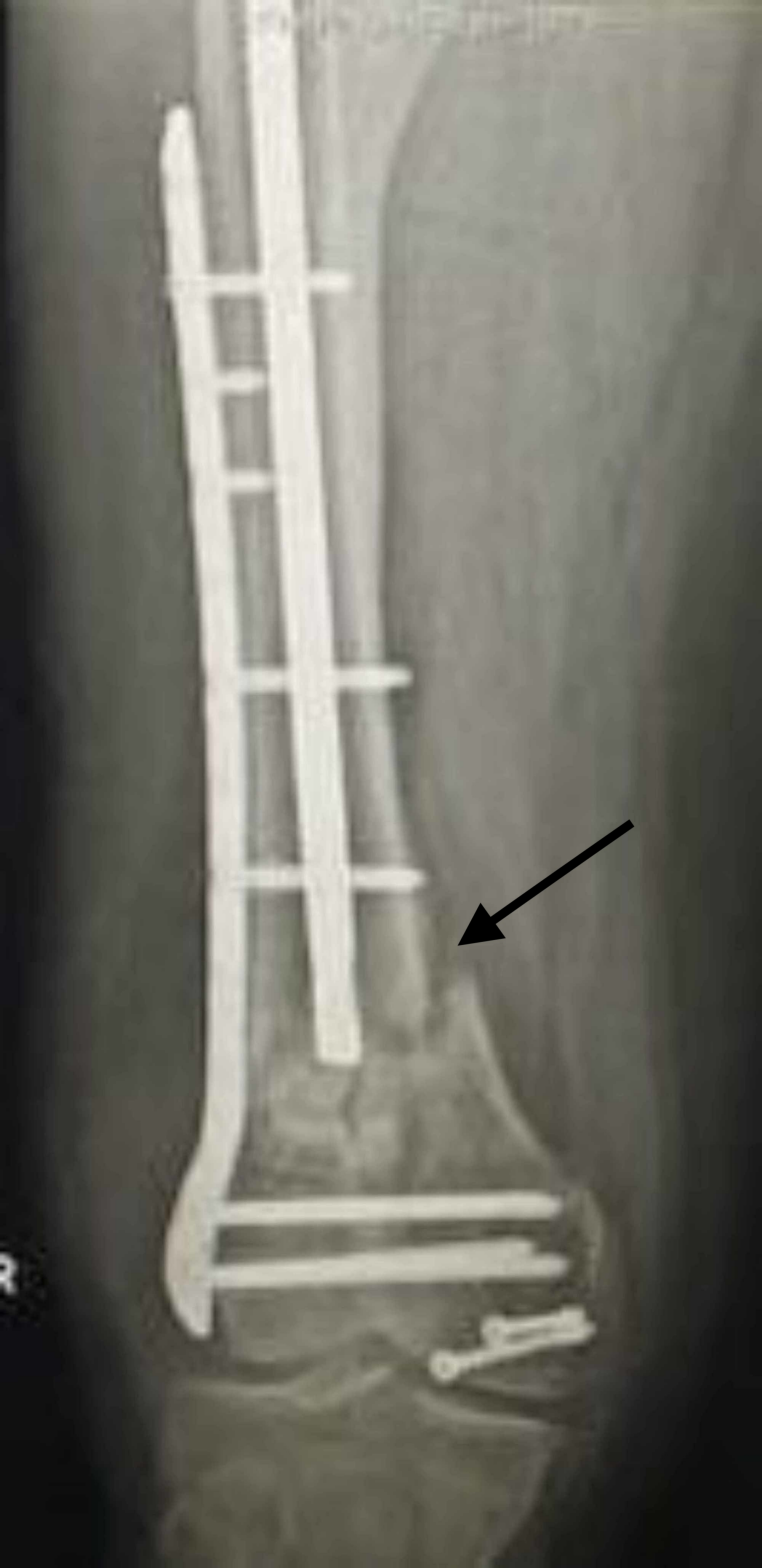
Non-union after single lateral plate fixation.

**Figure 4:**
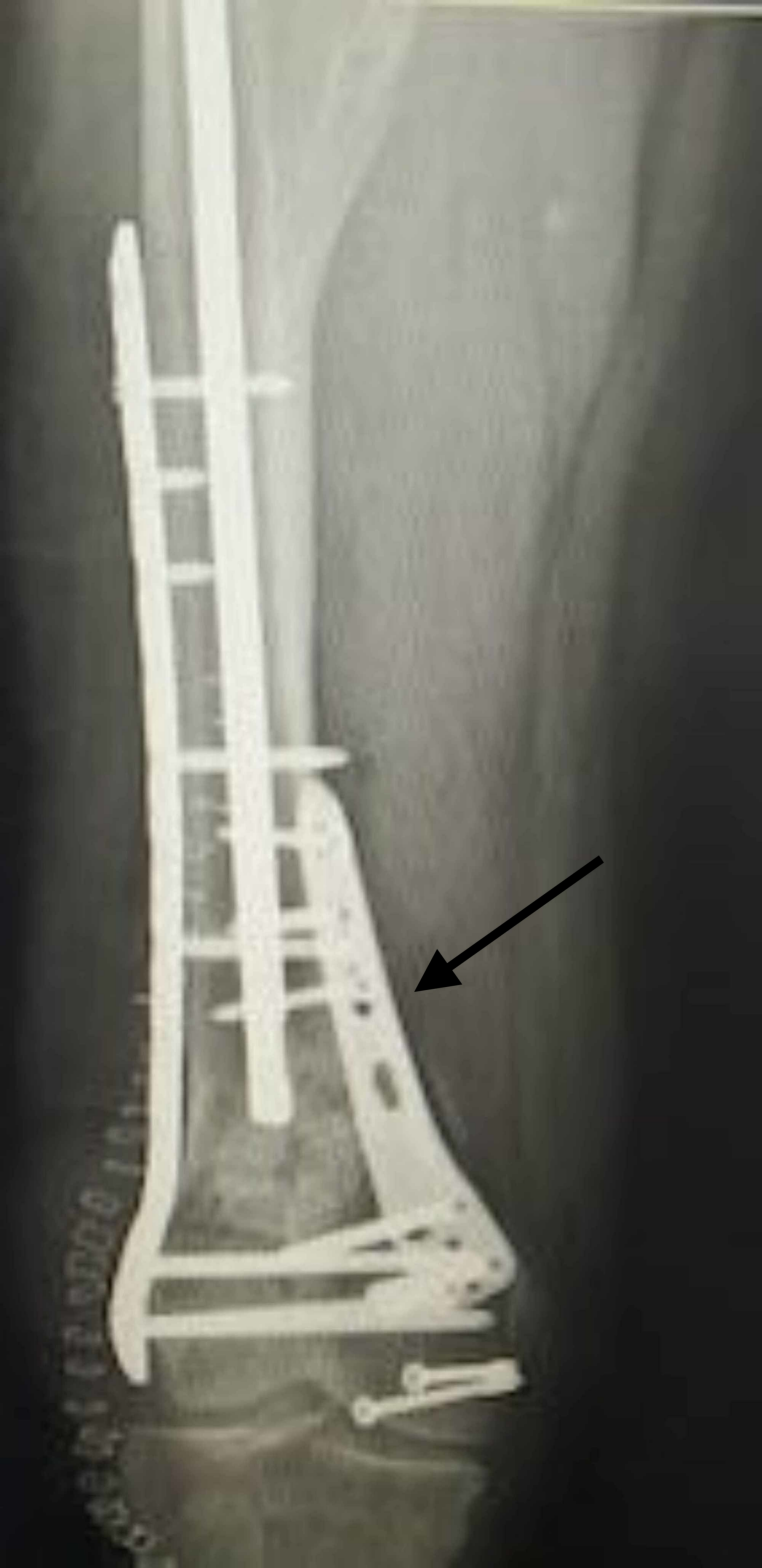
Revision with medial plate augmentation and bone grafting.

There is an increased risk of non-union in patients with comminuted distal femur fractures treated with single lateral locking plate [18]. In these cases of non-union of distal femur definitive treatment involves stabilization of non-union site with an additional medial plate and bone-grafting at the non-union site [19]. Non-union with medial bone loss more than 2 cm is an indication for medial plate augmentation and bone grafting (Figures 3, 4) [15].

Poor Bone Quality

In patients with distal femur fracture with osteoporotic bone, there is a high incidence of failure of fixation with a single lateral plate due to poor purchase of screw in osteoporotic bone. Metwaly and Zakaria demonstrated that dual plating in osteoporotic distal femur fractures by a single incision offers the stability of fixation with resultant early mobility and accelerated rehabilitation [20]. In the study by Todorov et al., medial augmented LISS (less invasive stabilization system) plating was found to have higher union rates compared to conventional LISS plating in osteoporotic distal femur fractures [21]. Steinberg et al. also found higher union rates with dual plating in fractures with osteoporotic bone [2].

Comminuted Distal Femur Fractures (AO type C3)

**Figure 5:**
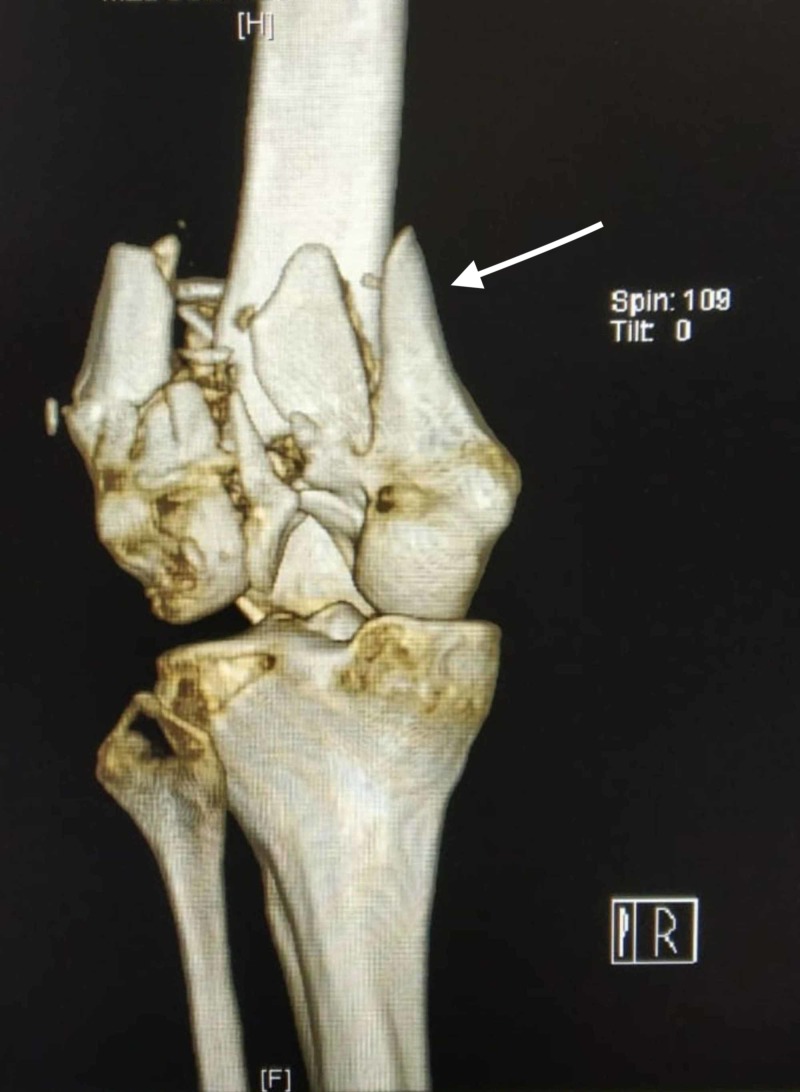
3D CT image showing a comminuted distal femur fracture in a 35-year-old male patient.

**Figure 6:**
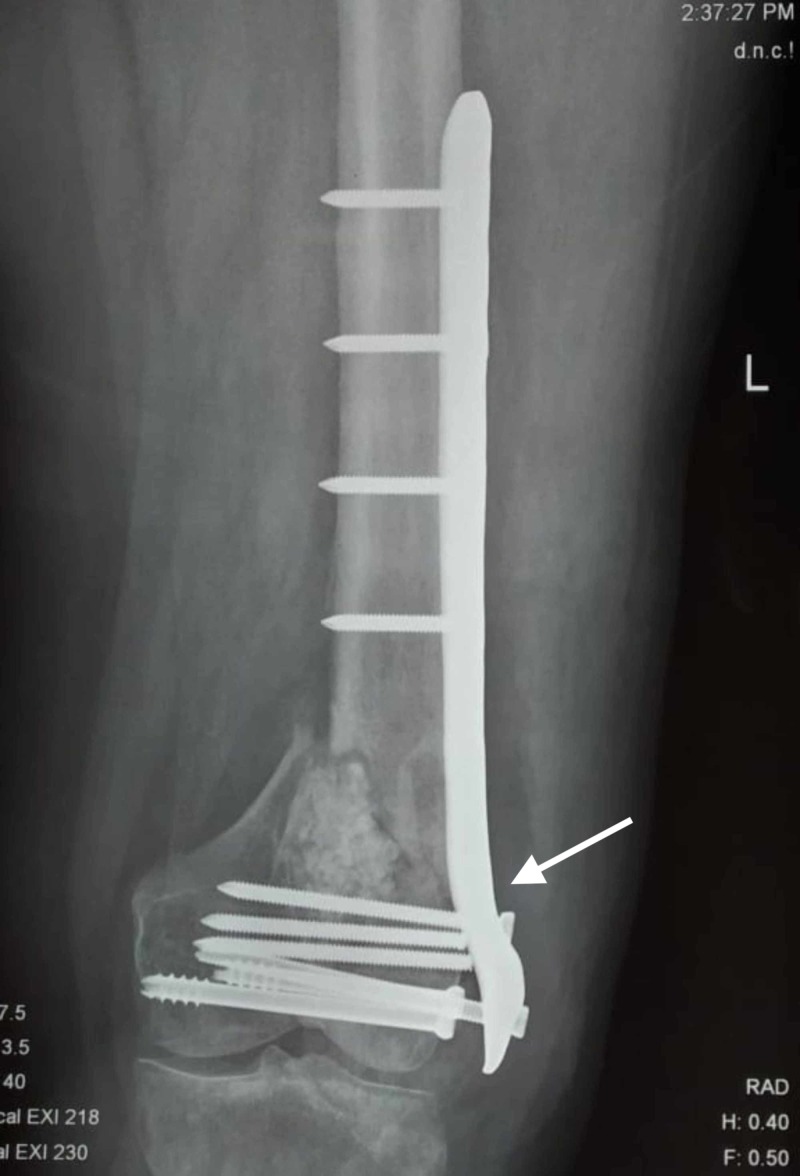
Failure following single lateral plate fixation.

**Figure 7:**
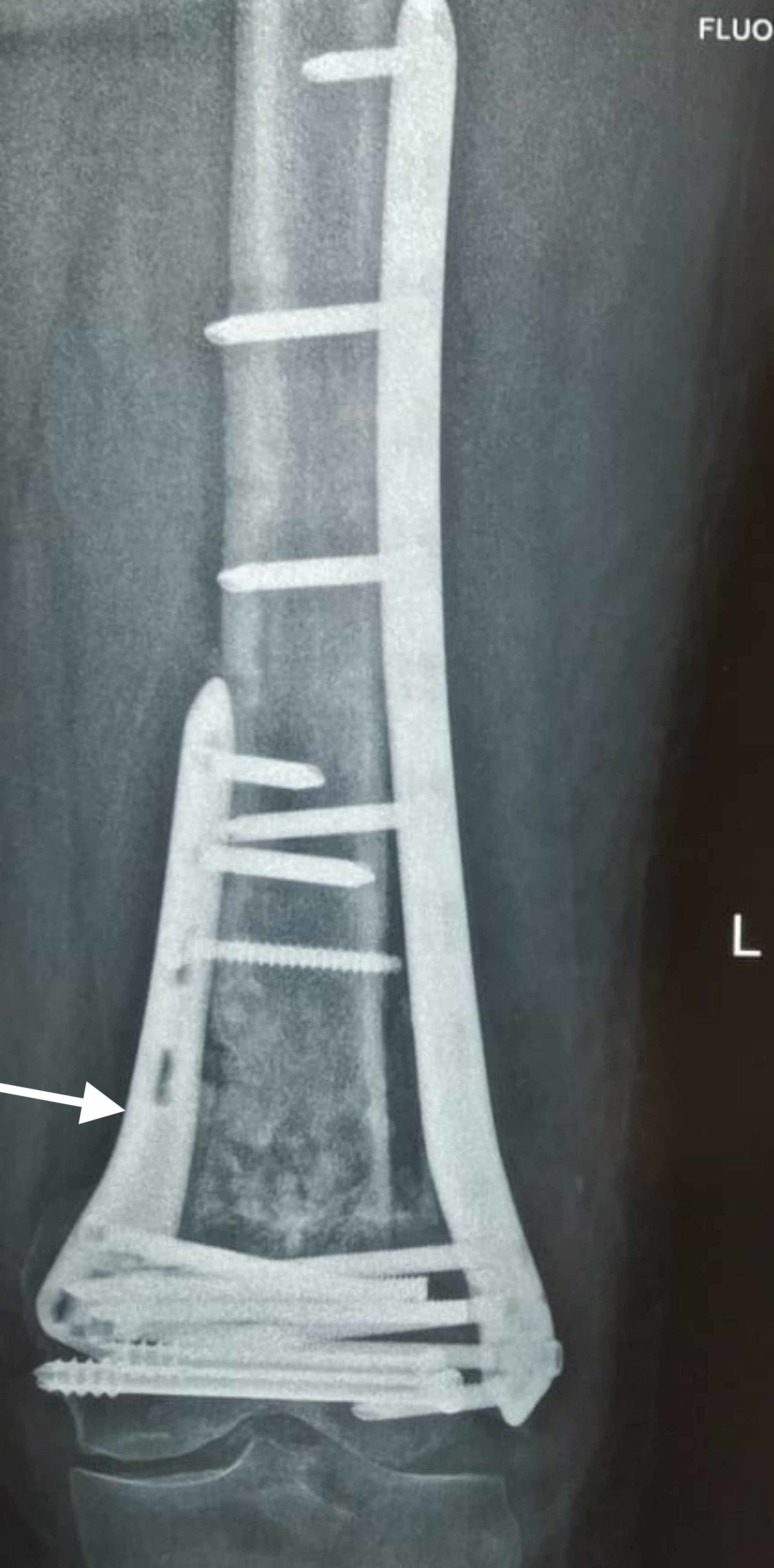
Revision with dual plating and bone grafting was done.

**Figure 8:**
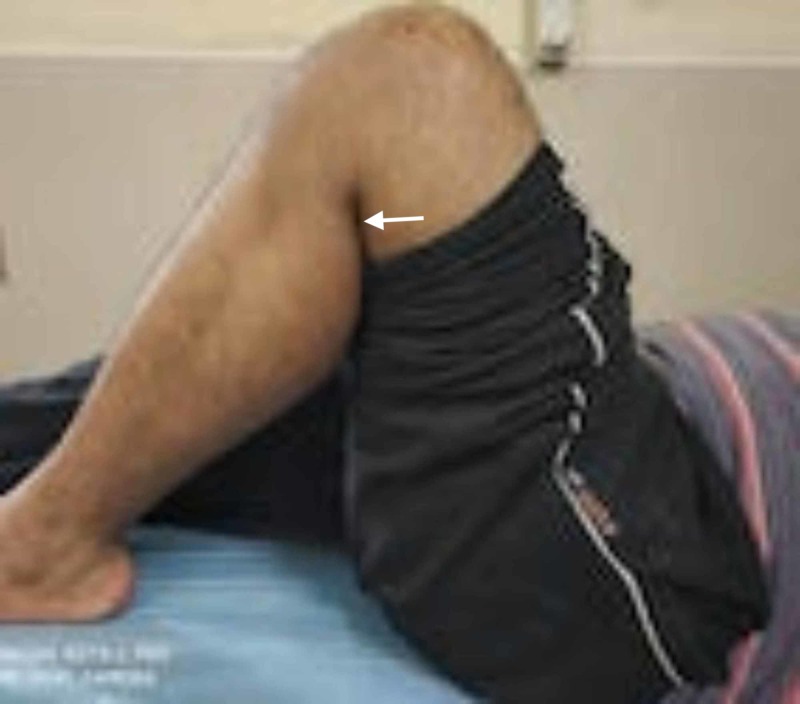
Knee flexion beyond 90 degrees in the follow-up.

In patients with AO type C3 distal femoral fractures, dual plating offers a more stable construct. Imam et al. demonstrated that dual plating fixation using anterior approach for type C3 distal femoral fractures is an efficient and safe method of management. It has several advantages such as precise exposure, easy manipulation, anatomical reduction, and stable fixation [1]. Steinberg et al. demonstrated higher rates of union with dual plating in AO type C3 distal femur fractures (Figures 5-8) [2].

Surgical technique

Position

The patient is positioned supine on a radiolucent operating table with the knee flexed at 30 degrees. Tourniquet use depends on the surgeon and may be used in very distal fractures. The use of a lateral femoral distractor with pins placed in the femoral diaphysis and proximal tibia metaphysis facilitates fracture alignment and disimpacts intra-articular fragments. Intra-operative use of C-arm is necessary for proper plate placement and fracture reduction and alignment [5].

Approach

**Figure 9:**
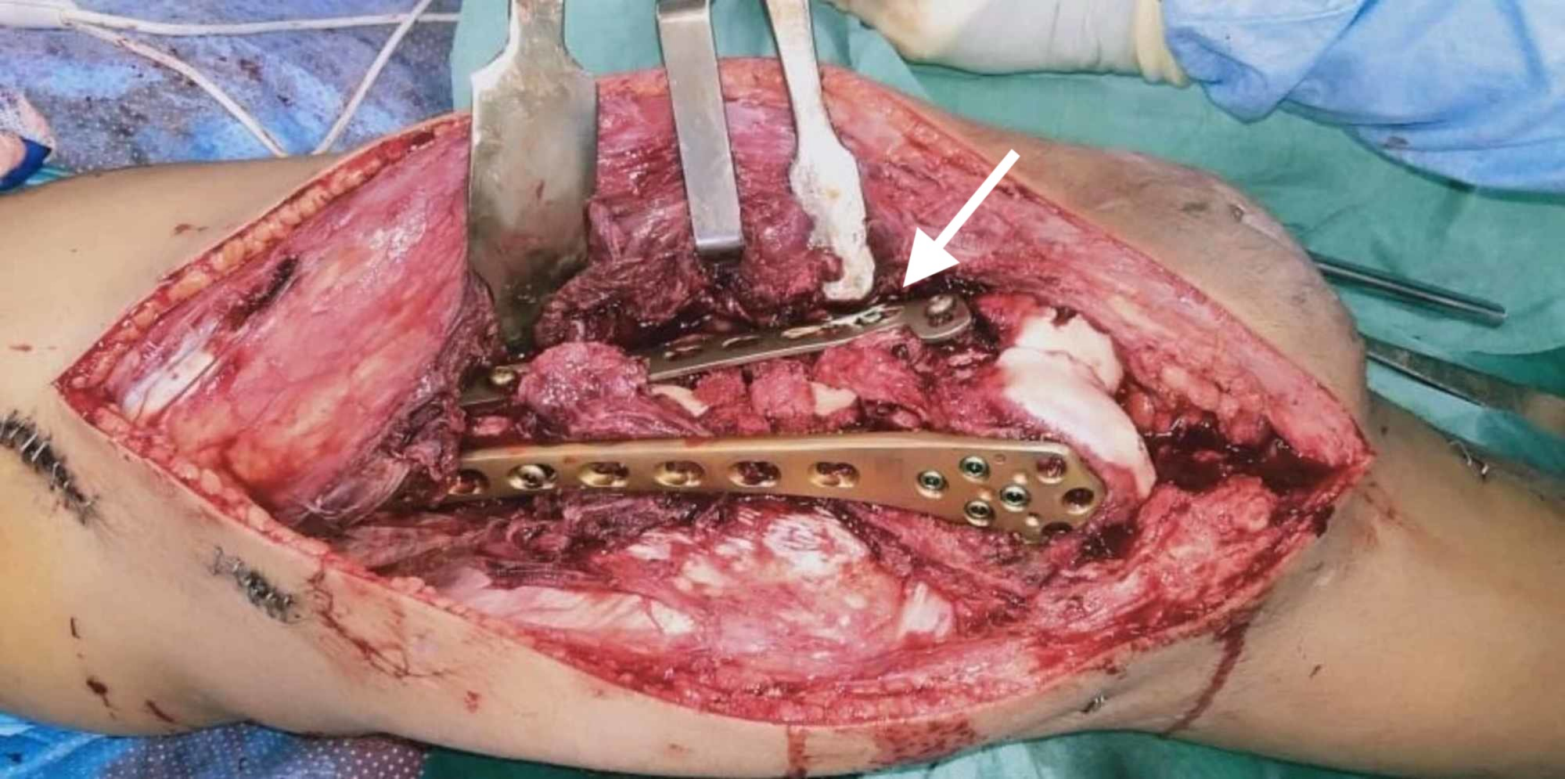
Single-incision lateral para-patellar incision used for the dual-plating distal femur. Also, note the orthogonal plating configuration used.

The choice of approach depends on the surgeon. There are two approaches, the dual incision approach and the single incision approach. The dual incision approach involves placing a medial and lateral incision for exposure of fracture and articular surface [2]. The single incision approach involves the use of a single para-patellar incision (lateral or medial) for exposure of fracture and articular surface (Figure 9). Imam et al. used a single midline incision and an extended medial para-patellar approach for dual plating of type C3 distal femoral fractures [1].

Precautions

Soft tissue attachments to bone fragments should be preserved to prevent any devascularisation of bone fragments. Soft tissue retractors should be used with care [5].

Choice of Implant

Dual 4.5 anatomically contoured locking plates are generally used. The T-buttress plate can also be used for medial condyle fixation. Cancellous screws of 6.5 mm are preferred for intercondylar fragment fixation [5]. According to the study by El Beaino et al. dual locking plates had higher torsional stiffness than conventional non-locking plates [22].

Plate Configuration

Orthogonal dual plate configuration provides more stable fixation and is biomechanically superior to dual adjacent plating for constructs fixed with either standard compression or locking plates [22].

Post-operative care

Early mobilization in a protected hinged knee brace should be done. Weight-bearing is to be allowed according to radiographic and clinical examination findings at the subsequent follow-up visits. Radiographic healing is established by the union of three cortices of the bone on the anteroposterior (AP) and lateral radiographic views of the bone. Clinical healing is confirmed by the absence of pain either with weight-bearing or with the application of stress over the injured area on examination. Malrotation is detected clinically by comparing the injured side to the normal side. Follow-up is to be continued until fracture healing and full weight-bearing [2].

## Conclusions

Dual plating of distal femur fractures offers a reliable stable fixation in cases with medial supracondylar bone loss, low trans-condylar bicondylar fractures, medial Hoffa fracture, peri-prosthetic distal femur fractures, non-union after failed fixation with single lateral plate, poor bone quality and comminuted distal femur fractures (AO type C3). Single-incision or dual incision approach may be used. Orthogonal plate configuration with locked plates provides stable fixation and allows for early rehabilitation. Early mobilization is necessary to prevent joint stiffness.
